# Surgical trainee research collaboratives in the UK: an observational study of research activity and publication productivity

**DOI:** 10.1136/bmjopen-2015-010374

**Published:** 2016-02-04

**Authors:** Aimun A B Jamjoom, Pho N H Phan, Peter J Hutchinson, Angelos G Kolias

**Affiliations:** 1 Centre for Clinical Brain Sciences, University of Edinburgh, Edinburgh, UK; 2 Medical School, University of Warwick, Coventry, UK; 3 Division of Neurosurgery, Addenbrooke's Hospital & University of Cambridge, Cambridge, UK; 4 Surgery Theme, Cambridge Clinical Trials Unit, Cambridge University Hospitals NHS Foundation Trust, Cambridge, UK

**Keywords:** Trainee Research Collaborative, Bibliometrics, Publication productivity

## Abstract

**Objectives:**

To analyse the research activity and publication output of surgical trainee research collaboratives in the UK.

**Setting:**

Surgical trainee research collaboratives in the UK.

**Participants:**

A total of 24 collaboratives were included in this study from 33 identified organisations. We excluded one group that focused purely on systematic review of the literature and eight groups for which we could not identify suitable data sources (website or trainee committee contact).

**Primary and secondary outcome:**

Primary data-points were identified for each collaborative including surgical subspeciality, numbers and types of projects. For published articles, secondary outcomes including study population size, journal impact factor, number of citations and evidence level were collected.

**Results:**

A total of 24 collaboratives met our inclusion criteria with a portfolio of 80 projects. The project types included audit (46%), randomised clinical trial (16%), surveys (16%), cohort studies (10%), systematic reviews (2.5%) and other or unidentifiable (9.5%). A total of 35 publications were identified of which just over half (54%) were original research articles. The median size of studied population was 540 patients with a range from 108 to 3138. The published works provided a varied compilation of evidence levels ranging from 1b (individual RCT) to 5 (expert opinion) with a median level of 2b (individual cohort study). The West Midlands Research Collaborative had the highest number of publications (13), citations (130) and h-index (5).

**Conclusions:**

The experience of UK-based trainee research collaboratives provides useful insights for trainees and policymakers in global healthcare systems on the value and feasibility of trainee-driven high quality surgical research.

**Strengths and limitations of this study**

- The study provides the most comprehensive analysis of the research activity and publication productivity of surgical trainee research collaboratives in the UK.
- It highlights the growing contribution and impact of an emerging paradigm of healthcare research.
- It provides insights for trainees and policymakers in global healthcare systems on the value and feasibility of trainee-driven high quality surgical research.
- Despite taking a systematic approach to searching for collaboratives there is a chance that the study may have missed some organisations.
- The study was limited to data found on the collaborative websites which could not be guaranteed to be up to date and our response rate for data clarification was 33%.

## Introduction

In the past 7 years, there has been a surge in the number and activities of trainee-led surgical research collaboratives across the UK. These groups have brought together trainees and medical students across the country with the aim of undertaking collaborative multicentre research projects. Trainee collaboratives are not a novel concept—the Royal College of Surgeons of General Practitioners arranged a 2-year trainee collaborative study looking at measles in the mid-1980s^1^. However, the contemporary incarnation of trainee collaboratives in the UK are modelled on regional general surgical trainee networks of which a notable example is the West Midlands Research Collaborative (WMRC). Impressively, the group completed recruitment for a multicentre randomised controlled trial (RCT) looking at the effectiveness of a wound protection device ahead of schedule^2^.

Surgical trainee collaboratives are organisations or groups, primarily run by trainees or medical students, which undertake multicentre patient-based surgical research. The recent expansion in the number of collaboratives throughout the UK has been driven by the wealth of trainee enthusiasm to participate in research and the recognition that collectively trainees are well positioned to answer important clinical questions. This is particularly evident with acute and emergency surgical care where trainees play a primary role in care provision. As the number of collaboratives increased across the country they have also started to work together, recently completing a National Appendicectomy Audit which included 3326 patients from 95 centres^3^. To coordinate these national projects the National Research Collaborative (NRC), an umbrella organisation, was established to facilitate multicollaborative networks and promote participation among a wide range of specialities. To help promote this, guides on how to set up a collaborative have been published focusing on the key structural and administrative principles^4^. At the heart of this lies an active trainee-led committee, effective communication, endorsement of national medical bodies and clear recognition for trainee participation.

With the successes and expansion of the trainee research collaboratives there has been a great deal of commentary on their value and importance^5^ ^6^. However, to date, there has been no objective analysis of the research activity and productivity of the trainee groups to help establish their true impact, guide their future direction and inform healthcare services in other countries. In this study, we aim to assess the current landscape of surgical trainee collaboratives in the UK, examine their activities and quantify their scientific impact through the systematic analysis of their publication record.

## Methods

We defined a surgical trainee research collaborative as an organisation or group, primarily run by trainees or medical students, which undertakes multicentre patient-based surgical research. We excluded groups that focused purely on systematic review of the literature or collaboratives for which we could not identify suitable data sources (website or trainee committee contact). To determine the number of collaboratives, we undertook a systematic online search using a range of key phrases including ‘trainee research’, ‘trainee collaborative’ and ‘surgical trainee research collaborative’ in March 2015. We also assessed lists of collaboratives posted on the NRC and Association of Surgeons in Training (ASiT) website^7^ ^8^. Identified collaboratives had their websites interrogated and committee contacted for a range of data-points including: surgical subspeciality, year of establishment, the number and type of projects. Two emails (3 weeks apart) were sent to the collaboratives between March and April 2015.

To establish the scientific impact of the trainee collaboratives, we assessed the publication record of the individual groups in April 2015. Publications were identified from collaboratives’ website listings and PubMed searches using the collaborative and project names. We included all PubMed-indexed publications, and excluded conference abstracts and proceedings. In the case of publications that emerged from multicollaborative work we allocated the article to the primary research group. Eligible publications were subsequently assessed for journal impact factor (IF), date of publication, number of authors and collaborators and number of citations received as per Google Scholar in April 2015.

As part of our analysis, we also applied two known academic metrics to individual collaboratives with publications: the h-index and m-quotient^9^. These metrics are used to assess individual scientists, however, we chose to apply them to each collaborative as a means to quantify and compare their academic productivity. The h-index is defined as the number of publications produced by an individual with at least that many citations. The m-quotient is the h-index divided by the number of years since the first publication. The m-quotient is used to give weight to temporal productivity and reflects positively on younger researcher. To assess the evidence emerging from trainee collaborative research, two independent investigators applied the Oxford Centre for Evidence Based Medicine ‘Levels of Evidence’ hierarchy to applicable articles^10^. This measure ranks evidence from 5 (lowest—expert opinion without critical appraisal) to 1a (highest—systematic review of RCTs).

## Results

**Figure 1:**
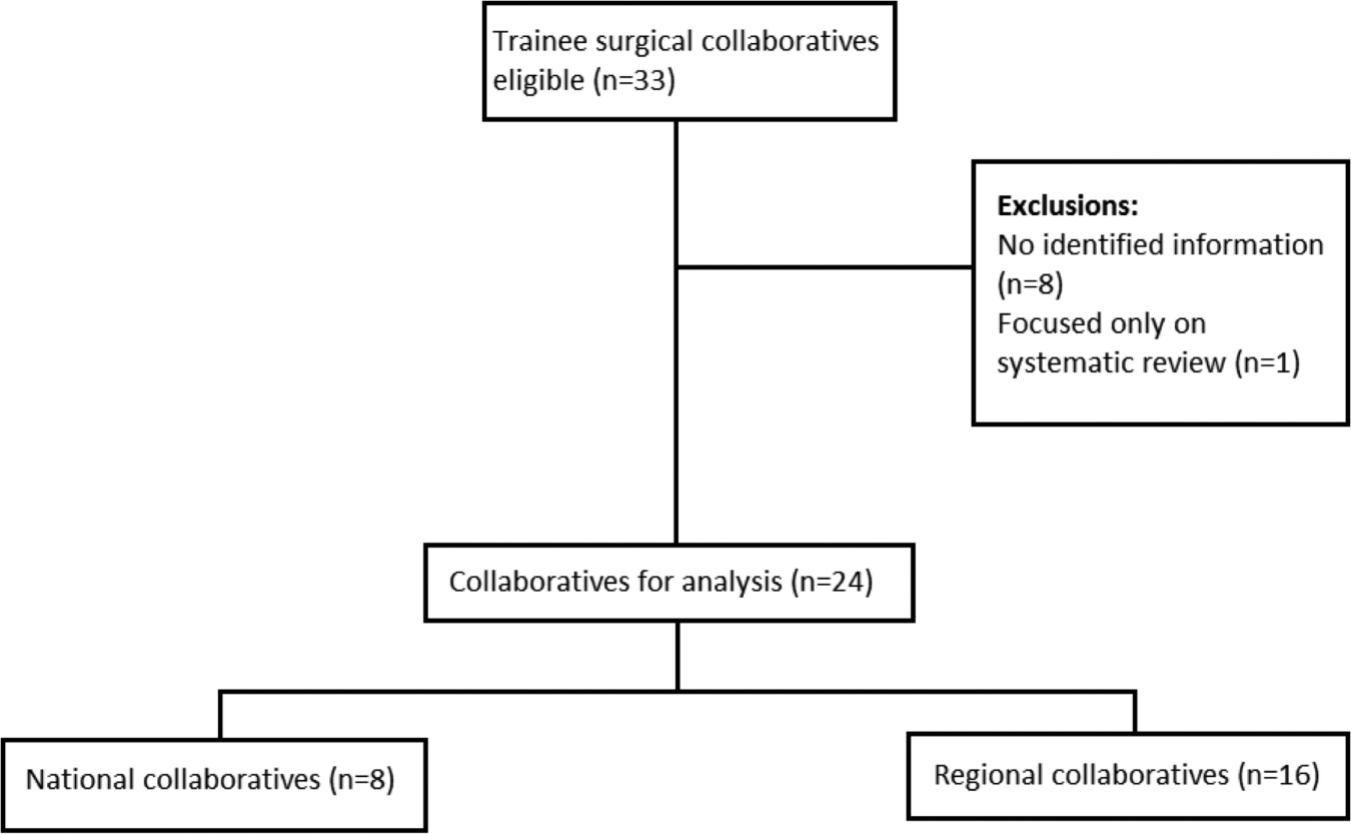
Flow diagram of search findings.

**Figure 2:**
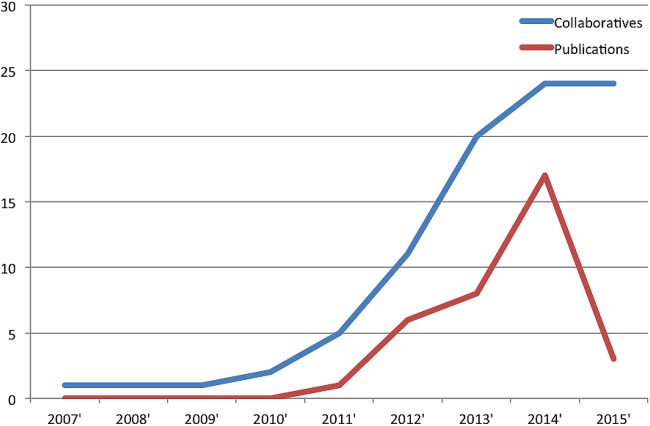
Number of collaboratives established and publications per annum (data collected in March 2015). The number of collaboratives is a cumulative value while the publications is a year-on-year number.

We identified a total of 33 organisations of which 24 met our criteria of a surgical trainee research collaborative (figure 1). The excluded nine groups contained one collaborative (Academic Surgical Collaborative) that focused purely on systematic review and meta-analysis which meant it did not meet our inclusion criteria of undertaking patient-based clinical research. We identified the other eight groups in lists of collaboratives on the NRC and ASiT websites but were unable to identify substantive data on the groups from websites or email contacts. From their names, these groups focused on orthopaedic surgery (2), general surgery (1), urology (1), obstetrics and gynaecology (1), cardiothoracic surgery (1) and unknown (2). We received 8 (33%) responses from the collaboratives alongside the data extracted from collaborative websites. Fourteen (58%) of the collaboratives focused on general surgical research, 2 (8%) on orthopaedic research and the remainder on a variety of surgical subspecialties including neurosurgery, transplant surgery, ENT surgery, cardiothoracic surgery, plastic surgery, paediatric surgery, vascular surgery and urology (table 1). Geographically, 16 (67%) concentrated on regional research while the remainder had a national remit. The year the groups were established ranged from 2007 to 2014. There was a major surge in the number of collaboratives between 2012 and 2013 with a total of 15 new collaboratives being established during this period (figure 2).

**Table 1:** Summary of surgical trainee research collaboratives in the United Kingdom

Speciality (n)	Name of collaborative	Year established	Total number of projects	Response to email
General surgery (14)	East Midlands Surgical Academic Network	2013	2	−
	London Surgical Research Group	2010	11	Yes
	Mersey Research Group for General Surgery	2012	3	−
	Northwest Research Collaborative	2012	4	−
	Severn and Peninsula Audit and Research Collaborative for Surgeons	2012	9	−
	Scottish Surgical Research Collaborative	2013	3	−
	Student Audit & Research in Surgery	2013	2	Yes
	South Yorkshire Surgical Research Group	2013	10	−
	Welsh Barbers Research Group	2011	3	Yes
	Wessex Surgical Trainee Research Collaborative	2013	3	Yes
	West Midlands Research Collaborative	2007	15	Yes
	Warwickshire Surgical Research Group	2012	4	−
	Yorkshire Surgical Research Collaborative	2014	1	−
	Irish Surgical Research Collaborative	2013	1	−
Orthopaedic surgery (2)	Collaborative Orthopaedic Research NETwork	2013	4	−
	Severn Audit & Research Collaborative in Orthopaedics	2014	1	−
Cardiothoracic surgery (1)	Cardiothoracic Trainees Research Collaborative	2011	5	−
ENT surgery (1)	ENT Trainee Research Collaboration	2013	2	Yes
Neurosurgery (1)	British Neurosurgical Trainee Research Collaborative	2012	5	Yes
Vascular surgery (1)	Vascular & Endovascular Research Network	2014	2	−
Urology (1)	Northern Urology Research Collaborative	2012	3	−
Plastic surgery (1)	Reconstructive Surgery Trials Network	2013	7	Yes
Paediatric surgery (1)	Paediatric Surgical Trainee Research Network	2011	2	−
Transplant Surgery (1)	Carrel Club Transplant Research Collaborative	2014	1	−

ENT, Ear, Nose and Throat surgery; JIF, journal impact factor.

### Research activities of surgical trainee collaboratives

In total, the collaboratives have worked on 80 projects of which 33 (41%) have been completed. Among the completed projects, there were 6 (18%) prospective national studies which increased to 12 (26%) among running projects and those under development. The predominant project type was clinical audit (46%) which involves the assessment of patient care against a predetermined standard. Major audits undertaken by trainee collaboratives include the national appendicectomy, chronic subdural haematoma and external ventricular drainage audit^3^ ^11^ ^12^. Other major project types included randomised trials (16%), surveys (16%), cohort studies (10%), systematic reviews (2.5%) and other or unidentifiable (9.5%). In total, trainee collaboratives have completed four RCTs throughout the UK and are currently driving the development and recruitment of a further nine clinical trials (table 2).

**Table 2:** List of Randomised Controlled Trials that surgical trainee research collaboratives have been involved with (this includes leading the trials or assisting with recruitment)

Clinical trials	
Reduction Of Surgical Site Infection using a Novel Intervention (ROSSINI trial)^2^	Trial to determine the effectiveness of wound edge protection devices in reducing surgical site infection after abdominal surgery. A total of 760 patients were enrolled ahead of schedule. The trial found there was no difference in wound infection between the two cohorts
Surgical Apgar Score in Clinical Practice (SAS trial)^13^	A pilot RCT randomising patients to routine postoperative care or an Apgar score influenced protocol
Dexamethasone Reduces Emesis After Major gastrointestinal Surgery (DREAMS trial)^14^	Trial assessing role of steroids in reducing emesis with patients randomised to 8 mg dexamethasone or placebo postoperatively
Reinforcement of Closure of Stoma Site (ROCSS trial)^15^	A trial assessing the placement of a biological mesh in order to reduce the rate of hernias at the site of stoma closure
Dexamethasone in Chronic Subdural haemtoma trial (Dex-CSDH trial)	A pragmatic randomised, double blind, placebo-controlled trial, clinical phase III study of a two-week course of dexamethasone for adult patients with a symptomatic chronic Subdural Haematoma
Randomised Evaluation of Surgery with Craniectomy for patients Undergoing Evacuation of Acute Subdural Haematoma(RESCUE-ASDH trial)^16^	RESCUE-ASDH is a multicentre, pragmatic, parallel group randomised trial that aims to compare the effectiveness of decompressive craniectomy vs craniotomy for the management of patients undergoing evacuation of an acute subdural haematoma
Peri-operative Recording of In apparent Myocardial Events (PRIME trial)	Trial to assess the effective of remote ischaemic preconditioning (RIPC) in improving outcome for patients undergoing major abdominal surgery
Melanoma Margins Trial (MelMarT trial)^17^	A Phase III, multicentre, multinational randomised control trial investigating 1 cm v 2 cm wide excision margins for primary cutaneous melanoma on disease recurrence and survival. Trainee collaborative helping with UK recruitment for this international RCT
Nail bed Injury Analysis trial (NINJA trial)^18^	A pragmatic multicentre study to assess whether the nail should be replaced or discarded after nail bed repair in children
Preheat Trial^19^	An RCT to assess the effective of ‘local heat preconditioning’ in reducing skin necrosis following reconstructive breast surgery
Hughes Abdominal Repair Trial (HART)^20^	Compares the current method of closing the abdominal muscles, with a ‘Hughes Repair’ in patients undergoing abdominal surgery for bowel cancer. The primary outcome is incisional hernia rates
Postoperative chlorohexidine for pneumonia (POP trial)^21^	Clinical trial assessing the effectiveness of oral decontamination with 0.2% cholorhexidine mouthwash on postoperative pneumonia rates
A Comparison of Post-Operative Pain Control using Epidural Vs a New Rectus Sheath Device	A clinical trial assessing the effectiveness of novel rectus sheath device for analgesia on postoperative pain

RCT, randomised controlled trial.

### Scientific impact of surgical trainee research collaboratives

A total of 35 publications were identified which consisted of research articles (54%), commentary/letters (11%), proposals (11%), protocols (11%), systematic reviews and meta-analyses (9%) and case reports (3%). Notably, the number of publications per year has risen steeply with 17 publications in 2014 preceded by 8 and 6 articles the prior 2 years, respectively (figure 2). For applicable research articles, we found that the median size of studied population was 540 patients with a range of 108–3138. In total, publications produced by the collaboratives have received 181 citations with a range of 0–39 citations. The journal IF ranged from no IF to 39.2 with a median of 2.1. We found that the number of authors on the publications ranged from 1 to 36 (median=7) while the number of collaborators (if present) ranged from 13 to 476 (median=207).

We compared collaboratives with a publication record in table 3 and found that the WMRC had the highest number of publications (13), citations (130) and h-index (5). WMRC was followed by the British Neurosurgical Trainee Research Collaborative and the Warwickshire Surgical Research Group. Among the nine collaboratives with publications, seven were general surgery focused. The median h-index for the general surgery groups was 1 (0–5), the neurosurgery group (4) and the paediatric surgery group (1). Applying the ‘Levels of Evidence’ hierarchy, the published works provided a varied compilation of evidence levels ranging from 1b to 5 with a median of 2b. We identified two published RCTs from trainee collaboratives including the ROSSINI trial and a pilot RCT looking at the use of the Surgical Apgar score to guide postoperative care.^2^ ^13^

**Table 3:** Academic metrics for the surgical trainee research collaboratives with publication record

Collaborative	Speciality	Publications, (n)	Median number of listed authors (range)	Median JIF	Total citations	h-index	m-quotient
West Midlands Research Collaborative	General surgery	13	8 (1–17)	3.28	130	5	1.25
British Neurosurgical Trainee Research Collaborative	Neurosurgery	7	12 (5–27)	0.95	27	4	1.3
Warwickshire Surgical Research Group	General surgery	4	4 (2–4)	1.22	12	1	0.33
London Surgical Research Group	General surgery	3	19 (5–36)	2.1	10	2	1
Student Audit & Research in Surgery	General surgery	2	3.5 (1–6)	22.21	1	1	1
Scottish Surgical Research Collaborative	General surgery	2	9.5 (7–12)	1.32	0	0	0
Severn and Peninsula Audit and Research Collaborative for Surgeons	General surgery	2	7.5 (4–11)	0.83	0	0	0
Paediatric Surgical Trainee Research Network	Paediatric surgery	1	5	5.21	1	1	1
Northwest Research Collaborative	General surgery	1	8	5.9	1	1	1

## Discussion

The landscape of UK trainee collaborative research has changed profoundly over the past 7 years. Our analysis demonstrates the value of grassroots trainee research with increasing numbers of projects and an expanding footprint in the literature. The assessment of the evidence levels also shows a respectable quality of research emerging from UK-based trainee collaboratives which, in selected cases, is impacting clinical practice. The success of the trainee collaboratives is based upon a number of connected factors. At its core, lies a highly motivated surgical trainee body working within a postgraduate training system which, in recent years, has put increasing weight on research experience. Importantly, this has been met with a marked shift in British surgical culture in promoting clinical research through a Royal College of Surgeons Clinical Trials initiative which has helped establish a network of trial centres throughout the UK.^22^

Our analysis suggests that the ambition of the groups is growing with more prospective national studies being undertaken and a mounting number of randomised trials under development. This shift, alongside the spike in the number of RCTs being conducted, is explained by increasing intercollaborative research and the emergence of national groups such as the British Neurosurgical Trainee Research Collaborative^23^. The NRC, a conglomeration of trainee research groups in the UK and Ireland, has played an important role in coordinating multicollaborative projects such as the National Appendicectomy audit^3^. The organisation has also been instrumental in promoting trainee collaboration in the UK and expanding the range of specialities partaking in collaborative trainee research. Recently, a number of regional anaesthetic groups have been established but we would like to see more specialities, particularly medicine, to start their own networks to help foster interspeciality projects.

Our analysis provides objective evidence of the current direction and activity within the UK trainee collaborative movement. Prior discussion had centred around the anecdotal achievements of single collaboratives which provided skewed impressions within the literature. Despite the trends towards more ambitious and larger studies, we also note important challenges for the trainee collaborative model. In particular, the recent intake of new collaboratives need to become fully established and start to produce a footprint within the literature. The NRC will play an important role in facilitating this process. Coupled to this, the growing ambition of the trainee collaboratives will require an increase in infrastructure to help facilitate their expansion. This includes access to centralised online databases and to statistical advice. Current arrangements for these facilities vary widely across collaboratives. Coordinating an open and affordable approach to database and statistician access would be a major achievement and help ensure the long-term success of the trainee collaborative movement. This would require working closely with a number of universities and clinical trials units to facilitate the necessary support for collaboratives that need it. However, at the core of the future success of the trainee collaborative model is maintaining enthusiasm through open participation and ensuring fair recognition of trainee involvement. Owing to the large numbers of involved trainees, the use of collaborator status on publications has become the method of choice for recognition. Our data highlight that this is happening with a total of 2528 collaborators on articles published by collaboratives.

## Study limitations

Our observational study has a number of limitations. Despite taking a systematic approach to searching for and identifying collaboratives with the UK, there is a chance that the study may have missed some organisation. Coupled to this, we were limited to data found on the collaborative websites which could not be guaranteed to be up to date. To try and overcome this, we contacted each collaborative with a set of questions to ensure we got a complete data set, however, we did not receive answers from 16 of the groups.

## Conclusions

Surgical trainees in the UK have been trailblazers in the development of a novel and effective model for healthcare research. Their experience provides useful lessons for trainees and policymakers in global healthcare systems on the value and feasibility of trainee involvement in delivering high quality clinical research. Wide spread availability of technological tools such as social media and centralised online databases has made establishing trainee collaboratives a comparatively easy and low cost exercise. This, coupled with senior support and clear authorship and collaboratorship criteria, provides the bedrock for a successful trainee collaborative^24^. Ultimately, we would like to see collaboratives, spanning all specialities, established in the UK and beyond working to improve patient care.
